# Interaction of *Acinetobacter baumannii* with Human Serum Albumin: Does the Host Determine the Outcome?

**DOI:** 10.3390/antibiotics10070833

**Published:** 2021-07-08

**Authors:** Camila Pimentel, Casin Le, Marisel R. Tuttobene, Tomas Subils, Krisztina M. Papp-Wallace, Robert A. Bonomo, Marcelo E. Tolmasky, Maria Soledad Ramirez

**Affiliations:** 1 Center for Applied Biotechnology Studies, Department of Biological Science, College of Natural Sciences and Mathematics, California State University Fullerton, Fullerton, CA 92831-3599, USA; camilapimentel99@csu.fullerton.edu (C.P.); thanhle1998@csu.fullerton.edu (C.L.); mtolmasky@fullerton.edu (M.E.T.); 2 Área Biología Molecular, Facultad de Ciencias Bioquímicas y Farmacéuticas, Universidad Nacional de Rosario, Rosario 2000, Argentina; mtuttobene13@gmail.com; 3 Instituto de Procesos Biotecnológicos y Químicos de Rosario (IPROBYQ, CONICET-UNR), Rosario S2002LRK, Argentina; tomassubils@gmail.com; 4 Research Service and GRECC, Louis Stokes Cleveland Department of Veterans Affairs Medical Center, Cleveland, OH 44106, USA; krisztina.papp@va.gov (K.M.P.-W.); Robert.Bonomo@va.gov (R.A.B.); 5 Departments of Medicine, Pharmacology, Molecular Biology and Microbiology, Biochemistry, Proteomics and Bioinformatics, Case Western Reserve University School of Medicine, Cleveland, OH 44106, USA; 6 CWRU-Cleveland VAMC Center for Antimicrobial Resistance and Epidemiology (Case VA CARES), Cleveland, OH 44106, USA

**Keywords:** *Acinetobacter baumannii*, antibiotic resistance, quorum sensing, biofilm, human serum, albumin

## Abstract

*Acinetobacter baumannii* has become a serious threat to human health due to its extreme antibiotic resistance, environmental persistence, and capacity to survive within the host. Two *A. baumannii* strains, A118 and AB5075, commonly used as model systems, and three carbapenem-resistant strains, which are becoming ever more dangerous due to the multiple drugs they can resist, were exposed to 3.5% human serum albumin (HSA) and human serum (HS) to evaluate their response with respect to antimicrobial resistance, biofilm formation, and quorum sensing, all features responsible for increasing survival and persistence in the environment and human body. Expression levels of antibiotic resistance genes were modified differently when examined in different strains. The *cmlA* gene was upregulated or downregulated in conditions of exposure to 3.5% HSA or HS depending on the strain. Expression levels of *pbp1* and *pbp3* tended to be increased by the presence of HSA and HS, but the effect was not seen in all strains. *A. baumannii* A118 growing in the presence of HS did not experience increased expression of these genes. Aminoglycoside-modifying enzymes were also expressed at higher or lower levels in the presence of HSA or HS. Still, the response was not uniform; in some cases, expression was enhanced, and in other cases, it was tapered. While *A. baumannii* AB5075 became more susceptible to rifampicin in the presence of 3.5% HSA or HS, strain A118 did not show any changes. Expression of *arr2,* a gene involved in resistance to rifampicin present in *A. baumannii* AMA16, was expressed at higher levels when HS was present in the culture medium. HSA and HS reduced biofilm formation and production of N-Acyl Homoserine Lactone, a compound intimately associated with quorum sensing. In conclusion, HSA, the main component of HS, stimulates a variety of adaptative responses in infecting *A. baumannii* strains.

## 1. Introduction

Infections caused by antibiotic-resistant bacteria have increased in frequency, resulting in significant patient morbidity and mortality [1,2]. *Acinetobacter baumannii* is a multidrug resistant (MDR) nosocomial pathogen that causes a variety of infections, many of them life-threatening, such as pneumonia, bacteremia, meningitis, and wound infections. *A. baumannii* strains resistant to carbapenems (CRAB) have frequently been reported [3], and this characteristic warranted the acknowledgemnt of CRAB as an “urgent threat” by the Centers for Disease Control and Prevention (CDC) in the 2019 Antibiotic Resistance Threats Report [4,5].

Few active antimicrobials are left to treat CRAB infections [6,7,8]. A laboratory- and population-based surveillance showed that most of the CRAB isolates also presented non-susceptibility to antibiotics generally considered “second-line” agents for treatment [9,10]. For example, ~94% demonstrated resistance against fluoroquinolones, both for levofloxacin and ciprofloxacin. Around 80% of the isolates possessed extendeded-spectrum β-lactamases (ESBLs), with 97% resistant to piperacillin/tazobactam and between 84 and 88% resistant to cefepime and ceftazidime. In contrast, tigecycline and colistin demonstrated resistance rates of approximately 40% and 7%, respectively [9,10]. However, these agents have significant limitations, such as a narrow therapeutic range (colistin, tigecycline), insufficient levels of free antibiotic in blood (colistin, tigecycline), significant adverse effects (colistin, tigecycline), and limited or no bactericidal activity (tigecycline) [10,11,12]. It is worth noting that there is only one orally administered antibiotic, minocycline, currently in use to treat CRAB infections. This not only limits treatment options but also complicates therapy by the need for hospitalization [10,12].

In addition to resistance, *A. baumannii* possesses intrinsic versatility in response to changing environments, within and outside the human host, and to acquire or change the expression of resistance and virulence genes [13,14,15,16,17,18,19]. When exposed to different human fluids or proteins, *A. baumannii* can modify the expression of a wide variety of genes that are needed to persist and survive [15,16,19,20,21,22]. Also, antibiotics can trigger DNA acquisition and affect the expression of resistance genes (Le et al., 2021 submitted manuscript [23,24,25]).

We recently demonstrated that human serum albumin (HSA) increases natural transformation as well as expression of competence associated and carbapenem resistance genes in both susceptible and resistant strains (Le et al., 2021 submitted manuscript). Cells exposed to HSA in combination with meropenem increased their capability to uptake DNA. This mix was also associated to enhanced expression of competence-associated genes. Similar changes were induced in cells cultured in the presence of human serum (HS) (Le et al., 2021 submitted manuscript). All these features can contribute to the increase of antibiotic resistance. To further understand how *A. baumannii* responds to HSA and human serum (HS), we focused on non-carbapenem antibiotic resistance genes, quorum sensing, and biofilm formation.

## 2. Results and Discussion

### 2.1. HSA and HS Modulate the Expression of Antibiotic Resistance Genes from Different Antibiotic Families

To further evaluate the role of HSA and HS in the expression level of antibiotic resistance genes, we performed quantitative RT-PCR (qRT-PCR) assays using total RNA extracted from two *A. baumannii* model strains (A118 and AB5075), and three carbapenem-resistant strains (AB0057, AMA16, and ABUH702). Cells were cultured in LB, LB supplemented with 3.5% HSA, or cultured in 100% pooled normal HS.

**Figure 1:**
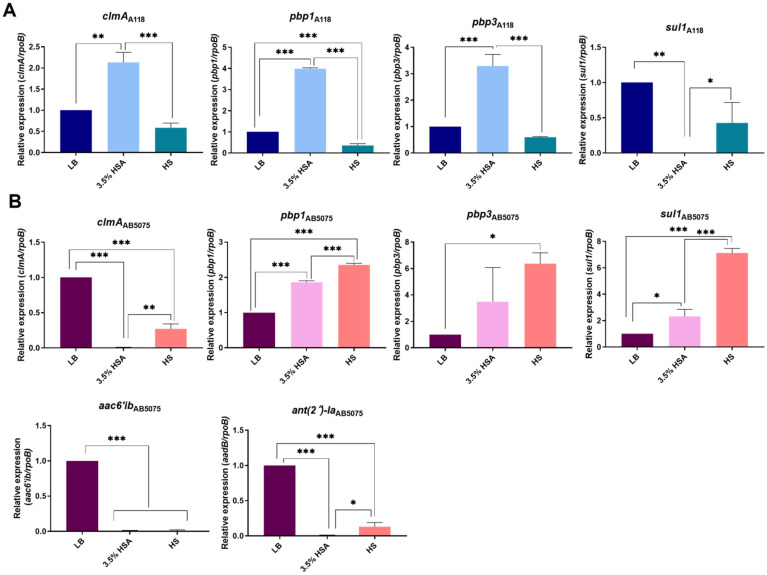
Effect of human serum albumin (HSA) and pooled normal human serum (HS) on the expression of antibiotic resistance genes of *A. baumannii* A118 and AB5075 strains. qRT-PCR of A118 (**A**) and AB5075 (**B**) strains genes associated with antibiotic resistance expressed in LB, LB supplemented with 3.5% HSA, or in HS. Fold changes were calculated using double ΔΔCt analysis. At least three independent samples were used. LB was used as the reference condition. Statistical significance (*p* < 0.05) was determined by ANOVA followed by Tukey’s multiple-comparison test, one asterisks: * *p* < 0.05; two asterisks: ** *p* < 0.01, and three asterisks: *** *p* < 0.001.

*A. baumannii* A118 showed an increase in expression of the chloramphenicol resistance *clmA* gene [26] when LB was supplemented with 3.5% HSA. Conversely, HS did not produce changes in the expression level of this gene (Figure 1A). In the case of *A. baumannii* AB5075 (Figure 1B) and AB0057 (Supplementary Figure S1), levels of expression of *clmA* were significantly down-regulated in LB plus 3.5% HSA and HS conditions. In contrast, in the *A. baumannii* AMA16 and ABUH702 strains, *clmA* was up-regulated when growing under these conditions (Figure S1).

**Figure 2:**
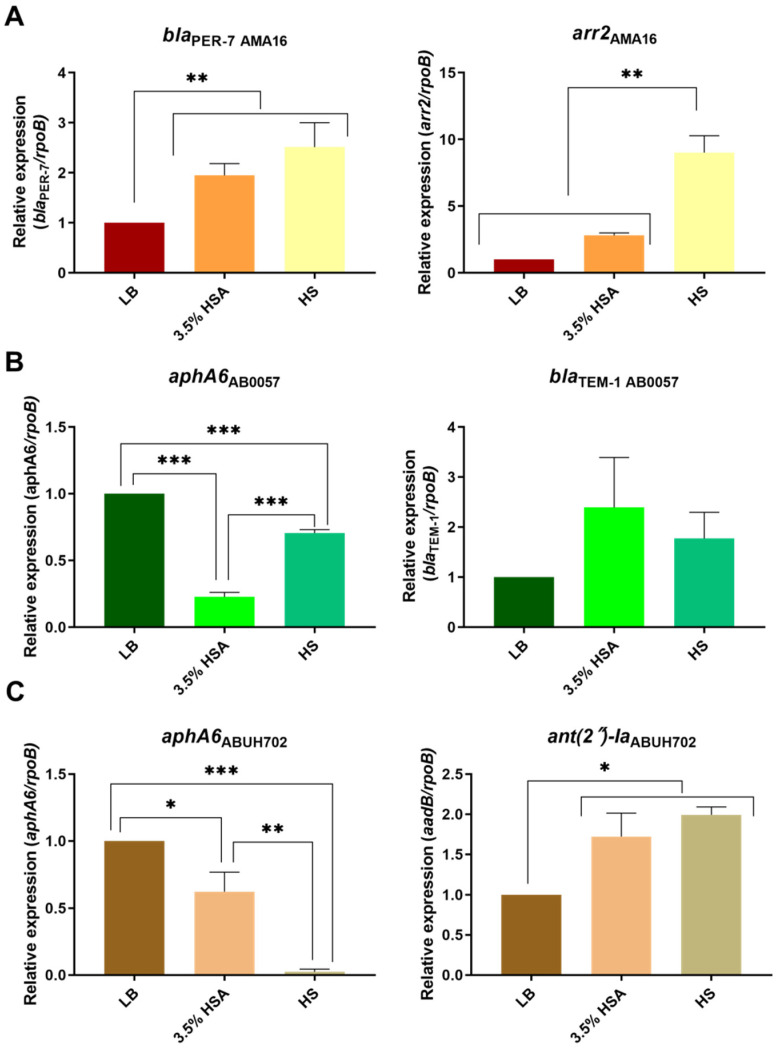
Effect of human serum albumin (HSA) and pooled normal human serum (HS) on the expression of antibiotic resistance genes of *A. baumannii* strains. qRT-PCR of AMA16: (**A**) AB0057 (**B**) and ABUH702 (**C**) strains genes associated with antibiotic resistance expressed in LB, LB supplemented with 3.5% HSA, or in HS. Fold changes were calculated using double ΔΔCt analysis. At least three independent samples were used. LB was used as the reference condition. Statistical significance (*p* < 0.05) was determined by ANOVA followed by Tukey’s multiple-comparison test, one asterisks: * *p* < 0.05; two asterisks: ** *p* < 0.01; and three asterisks: *** *p* < 0.001.

We previously observed that the expression levels of carbapenem-resistance genes were affected when LB was supplemented with 3.5% HSA or HS (Le et al., 2021 submitted). Further examination of the effect of these supplements on other genes related to β-lactam resistance, showed that the expression levels of *pbp1* and *pbp3*, coding for penicillin-binding proteins [27,28], were up-regulated in LB supplemented with 3.5% HSA in *A. baumannii* A118, AB5075 (Figure 1A,B), and ABUH702 (Figure S1). All strains but A118 underwent an increase in levels of expression of *pbp1* and *pbp3* when cultured in HS (Figure 1, Figure 2 and Figure S1). The *bla*_PER-7_ gene, present only in strain AMA16, was also up-regulated in the presence of 3.5% HSA or HS (Figure 2A).

**Figure 3:**
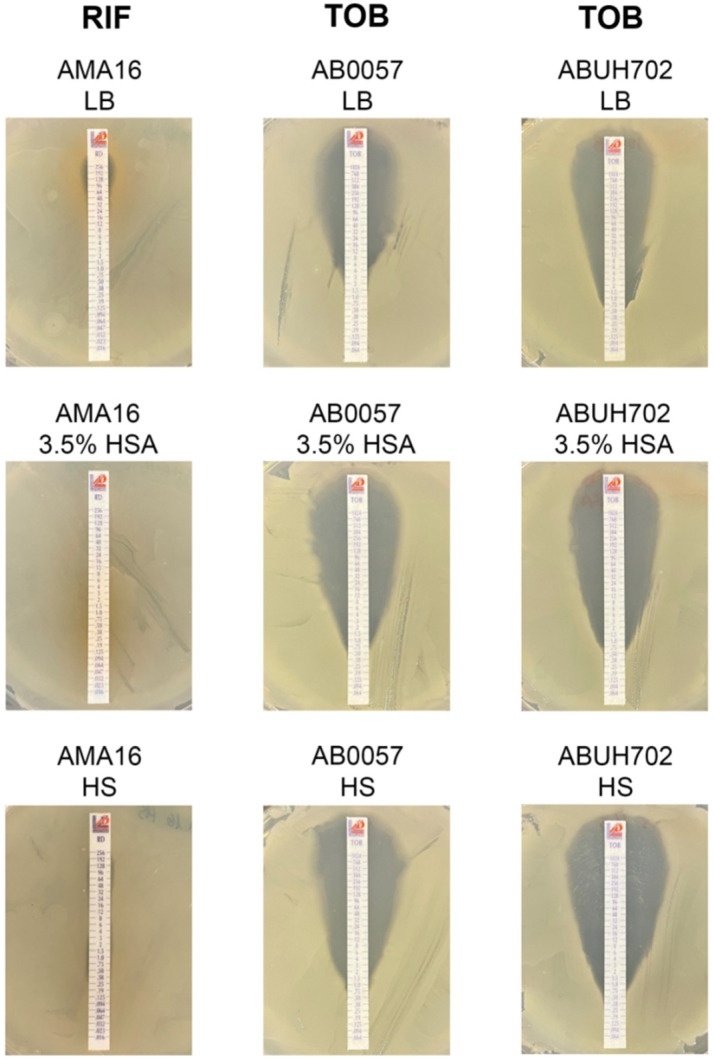
Effect of human serum albumin (HSA) and pooled normal human serum (HS) on the antimicrobial susceptibility of *A. baumannii* strains. AMA16, AB0057, and ABUH702 strains grew in LB broth, LB broth plus 3.5 % HSA, or in HS were used to performed rifampicin (RIF) and tobramycin (TOB) susceptibility. Minimum inhibitory concentration (MIC) was performed by E-test (Liofilchem, Italy) following CLSI recommendations.

*A. baumannii* AB5075 strain is resistant to most aminoglycoside antibiotics (gentamicin, amikacin and tobramycin (Table 1) and posseses the *aac(6a)-Ib* and *ant(2n)-Ia* (*aadB*), which encode an aminoglycoside 6′-N-acetyltransferase and an aminoglycoside O-nucleotydyltransferase, respectively [29]. qRT-PCR revealed a significant reduction in the expression of both genes in the presence of 3.5% HSA or HS (Figure 1B). In contrast, *ant(2n)-Ia*, also present in *A. baumannii* ABUH702, was expressed at significantly higher levels in cells cultured in LB supplemented with 3.5% HSA or HS (Figure 2C). The *armA* gene present in *A. baumannii* AMA16, which encodes a 16S rRNA methylase that confers resistance to amikacin, plazomicin, gentamicin and tobramycin [30], did not exhibit any significant changes in the tested conditions (Figure S1). The expression of *aphA6*, a gene present in *A. baumannii* AB0057 and ABUH702 that codes for an aminoglycoside O-phosphotransferase [29], was significantly down-regulated in the presence of 3.5% HSA or HS (Figure 2B,C). This effect could be related to an increase observed in the susceptibility of *A. baumannii* AB0057 to tobramycin (Figure 3 and Table S1). However, we did not observe any decrease in the MIC values of amikacin, tobramycin, and gentamicin in the AB5075, AB0057 and ABUH702 strains (Figure 3 and Figures S2 and S3).

Modifications in levels of resistance to rifampicin, a drug that alone or in combination with colistin has been used for severe infections due to MDR *A. baumannii* [31], were also evaluated. An increase in the susceptibility to rifampicin was observed in the case of *A. baumannii* AB5075 in the presence of 3.5% HSA or HS, while changes were not observed in strain A118 (Table 1). In the case of *A. baumannii* AMA16, which posseses the *arr2* gene, related to rifampicin resistance, a significant increase in the expression level of *arr2* and a concomitant increase in MIC were observed when growing in HS (Figure 2A and Figure 3).

The sulfonamine resistance gene *sul1* [32], present in *A. baumannii* A118, AB5075, AMA16, and AB0057, responded differently to the additions. It was down-regulated when growing in medium supplemented with 3.5% as determined using qRT-PCR analysis in *A. baumannii* A118, AMA16, and AB0057 (Figure 1A and Figure S1). Conversely, this gene was expressed at higher levels in *A. baumannii* AB5075 exposed to 3.5% HSA or HS, and in *A. baumannii* AB0057 cultured in HS (Figure S1).

The impact of human fluids and/or human proteins on the expression of antibiotic resistance genes and the concomitant levels of resistance are largely unknown [18,22,33]. Previous work showed that 0.2% HSA and 4% Human Pleural Fluid altered the expression of genes related to antibiotic resistance [17,21]. Moreover, Huang et al. demostrated the ability of mucin to bind antibiotics, such as colistin, which leads to a reduction in the efficacy of antibiotics used to treat *A. baumannii* infections [34]. The results described in this analysis indicate that in five different strains, the expression levels of resistance genes are affected by HSA and HS. Resistant and susceptible strains behaved identically with respect to the β-lactam and sulfonamide resistance-associated genes, which were upregulated in the presence of 3.5% HSA. However, while the addition of 3.5% HSA to *A. baumannii* A118 cultures produced an increase in expression levels of a chloramphenicol resistance gene, the opposite effect was observed in carbapenem-resistant strains. For some genes or groups of genes encoding resistance to an antibiotic class, the effect in the expression levels varied depending on the strain and/or the condition. In the case of β-lactam resistance-associated genes, an increase in the expression was observed for most of the genes in all the strains in both conditions (Figure 1 and Figure 2). The aminoglycoside resistance genes were mostly down-regulated in all strains and conditions, with the only exception of *ant(2n)-Ia* in the ABUH702 strains exposed to 3.5% HSA or HS (Figure 1B and Figure 2C). Future studies will increase the understanding of how the processes used by bacteria to respond to the presence of human fluids and/or human proteins contribute to antibiotic treatment failure.

### 2.2. HSA and HS Negatively Affects Biofilm Formation

Since biofilm formation has a direct impact in antibiotic resistance [33,35,36,37], leading in some cases to treatment failure, we investigated the effect of 3.5% HSA and HS in the expression of genes involved in biofilm formation and the corresponding impact on phenotype.

**Figure 4:**
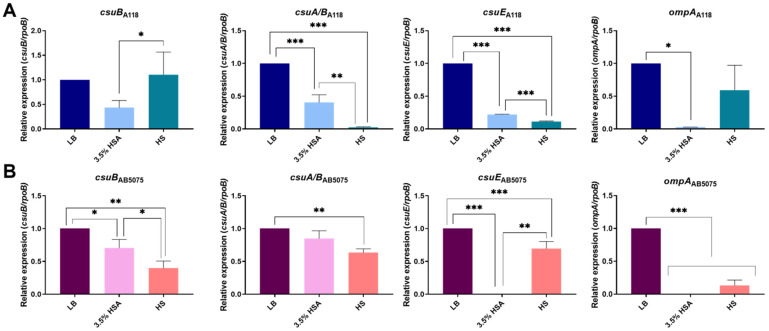
Effect of human serum albumin (HSA) and pooled normal human serum (HS) on the expression of biofilm formation genes in *A. baumannii* strains. qRT-PCR of A118 (**A**) and AB5075 (**B**) strains of the *csuB, csuA/B, csuE,* and *ompA* genes biofilm formation related in cells cultured in LB, LB supplemented with 3.5 % HSA, or in HS. Fold changes were calculated using double ΔΔCt analysis. At least three independent samples were used. LB was used as the reference condition. Statistical significance (*p* < 0.05) was determined by ANOVA followed by Tukey’s multiple-comparison test, one asterisks: * *p* < 0.05; two asterisks: ** *p* < 0.01 and three asterisks: *** *p* < 0.001.

qRT-PCR analysis of *csuA/B*, *csuB*, and *csuE* genes belonging to CsuA/BABCDE chaperone-usher secretion system [38] was carried out in A118 and AB5075 cells grown in LB, LB supplemented with 3.5% HSA, and HS (Figure 4). The transcriptional level of A118 and AB5075 *csuA/B*, *csuB*, and *csuE* showed a significant down-regulation under 3.5% HSA and HS conditions, with the exception of *csuB* for the A118 strain (Figure 4A,B). Moreover, analysis of mRNA extracted from *A. baumannii* AMA16, AB0057, and ABUH702 strains cultured in medium containing 3.5% HSA or in HS showed that the expression of *csuA/B* was significantly reduced in the presence of both conditions, with the exception of AB0057 strain grown in LB plus 3.5% HSA (Figure S4). In addition, the outer membrane protein A (OmpA) of *A. baumannii* is reported as playing a role in the development of robust biofilms on plastic [39]. The transcriptional analysis of *ompA* was also investigated and we observed a down-regulation in the presence of 3.5% HSA or HS with respect to LB condition for all strains with the exception of ABUH702 strain (Figure 4 and Figure S4).

**Figure 5:**
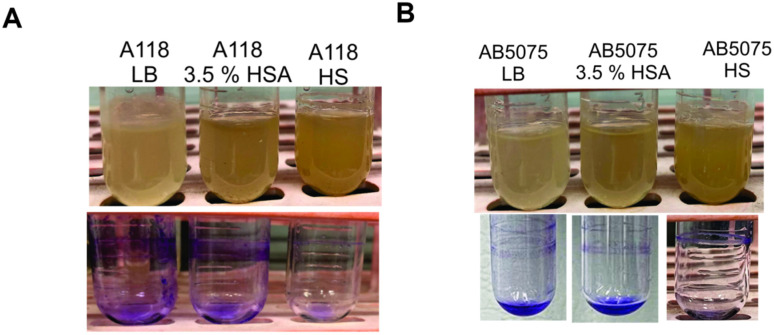
Effect of human serum albumin (HSA) and pooled normal human serum (HS) on biofilm formation in *A. buamannii*. Biofilm assays performed in A118 (**A**) and AB5075 (**B**) strains grown in LB, LB supplemented with 3.5% HSA, or in HS. Tubes were stained with 1% crystal violet.

These results were in agreement with biofilm formation in tubes, since a reduction or absence of biofilm was observed (Figure 5). To further examine the inhibitory effect of 3.5% HSA and HS on other *A. baumannii* strains, AMA16, AB0057, and ABUH702 strains were grown in LB, LB plus 3.5% HSA, or HS. Less biofilm production was observed supporting the previous observation (Figure S4). Our results showed a strong negative effect of HSA and HS in biofilm formation in all the included strains, leading us to conclude that this inhibitory effect occurs in the different *A. baumannii* strains.

A previous work analysing the biofilm formation of different *A. baumannii* isolates that recovered from blood or sputum, showed that blood isolates formed less biofilm compared to respiratory isolates [40]. Our results are in agreement with this observation [40]. Another human molecule that also exhibited biofilm formation inhibition in *A. baumannii* strains was L-Adrenaline [41]. In addition, a recent study reported that lactoferrin has antimicrobial and antibiofilm activities against *A. baumannii* clinical strains that were isolated from wounds, blood, urine, and sputum/bronchial wash [42]. Lactoferrin is an innate immune glycoprotein produced in high concentrations in both human and bovine milk, and is considered one of the most effective iron chelators. Lactoferrin’s binding affinity for iron is 300 times higher than that of transferrin. These observations provide evidence that indicates the complex interplay between this pathogen’s and both human and bovine isoforms of host products [42]. Together, these observations demonstrate how human proteins can have an important and significant impact on biofilm formation.

### 2.3. Quorum Network Is Altered by HSA and HS

Many bacteria communicate with each other and respond collectively to a changing environment through quorum sensing (QS) [43]. QS permits bacteria to monitor one another’s presence and to modulate gene expression in response to changes in population density, controlling survival and virulence [43,44].

**Figure 6:**
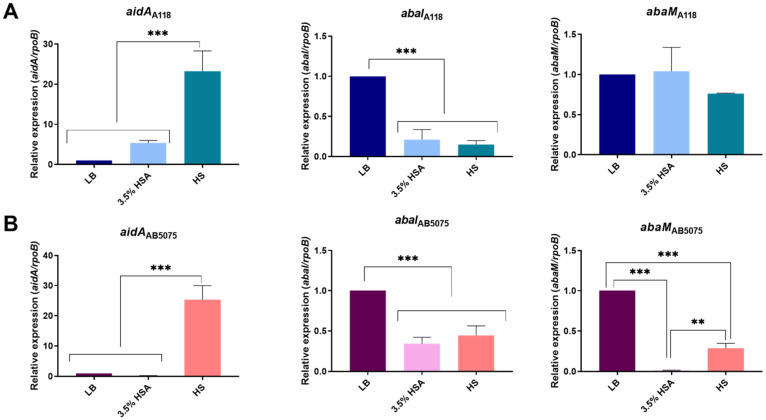
Effect of human serum albumin (HSA) and pooled normal human serum (HS) on the expression of quorum sensing genes in *A. baumannii*. qRT-PCR of A118 (**A**) and AB5075 (**B**) strains of the *aidA*, *abaI,* and *abaM* genes associated with quorum sensing and quorum quenching expressed in LB, LB supplemented with 3.5% HSA, or in HS. Fold changes were calculated using double ΔΔCt analysis. At least three independent samples were used. LB was employed as the reference condition. Statistical significance (*p* < 0.05) was determined by ANOVA followed by Tukey’s multiple-comparison test, two asterisks: ** *p* < 0.01 and three asterisks: *** *p* < 0.001.

**Figure 7:**
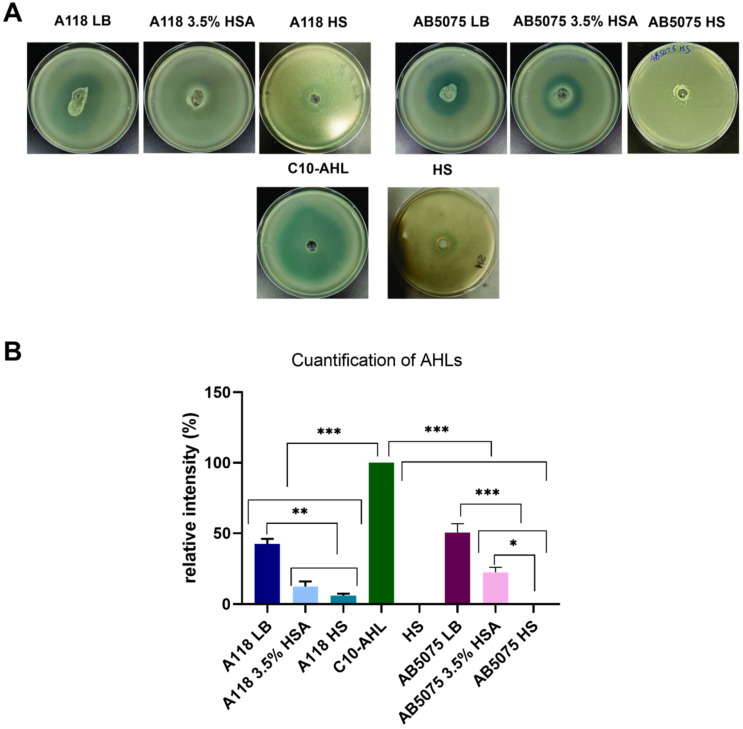
Effect of human serum albumin (HSA) and pooled normal human serum (HS) on quorum sensing in *A. baumannii*. (**A**) N-Acyl Homoserine Lactone (AHL) detection in *A. baumannii* A118 and AB5075 supernatants of bacterial cultured in LB, LB supplemented with 3.5% HSA, or HS, using *Agrobacterium tumefaciens* as reported strain. The presence of AHL was determined by the development of the blue color. (**B**) Quantification of 5,5′-dibromo-4,4′-dichloro-indigo was estimated as the percentage relative to C10-AHL standard, measured with ImageJ (NIH). As negative control, HS was used. The mean ± SD is informed. Statistical significance (*p* < 0.05) was determined by ANOVA followed by Tukey’s multiple-comparison test. one asterisks: * *p* < 0.05; two asterisks: ** *p* < 0.01 and three asterisks: *** *p* < 0.001.

To explore the QS network in *A. baumannii* A118 and AB5075 strains growing in LB plus 3.5% HSA or in HS, qRT-PCR analysis of important genes of QS/quorum quenching (*abaI, aidA,* and *abaM*) were carried out [45,46,47]. The results showed that *aidA* expression was up-regulated and *abaI* expression was down-regulated under both treatments in both strains (Figure 6). Differences were not observed in the expression levels of *abaM* between LB, LB plus 3.5% HSA, and HS in *A. baumannii* A118 (Figure 6A). In contrast, in *A. baumannii* AB5075, the levels of expression of *abaM* were significantly reduced under both treatments (Figure 6B). These results suggest that when *A. baumannii* is exposed to HSA, the cells modulate the expression of QS network genes to reduce AHL synthesis or increase its degradation. We confirmed these findings by using *A. tumefaciens* as a bacterial biosensor. Supernatants from *A. baumannii* A118 and AB5075 cultures in LB showed the highest AHL production, suggesting down-regulation of QS and/or up-regulation of quorum quenching in the presence of HSA molecules or HS components (Figure 7). These results are consistent with a previous observation that *A. baumannii* AB5075 cultured in human pleural fluid produced reduced levels of AHLs [16].

In addition, considering the role of the AHL molecules in the upregulation of *bfmS* and *bfmR* genes involved in robust biofilm formation on abiotic surfaces in *A. baumannii* [48], and the evidence that *abaI* mutants are impaired in biofilm development [49], our results agreed with reduced biofilm formation observed when the cells were exposed to 3.5% HSA and HS.

Taken together, the results described herein indicate that both strains coordinate and modulate the activity of many genes that regulate the response to a variety of environments and conditions. These responses likely play a major role in persistence, virulence, and colonization.

## 3. Materials and Methods

### 3.1. Bacterial Strains

The model *A. baumannii* strains A118 and AB5075, which show a different degree of susceptibility and virulence, were used [16,21,50,51]. Additional strains containing metallo β-lactamases, such as New Delhi metallo- β-lactamase (NDM) or oxacilinases (OXA) were used. The used strains were AMA 16 strain (NDM-1 positive strains) [52], ABUH702 (carbapenem resistance due to increase expression of *bla*_OXA-66_ by IS*Aba1*) [53], and AB0057 (*bla*_OXA-23_) [52].

### 3.2. RNA Extraction and Quantitative Reverse Transcription Polymerase Chain Reaction (qRT-PCR)

Overnight cultures of *A. baumannii* strains were diluted 1:10 in fresh LB medium; 3.5% HSA containing LB medium or pooled normal human serum (HS) and incubated with agitation for 5 h at 37 °C. Pure HSA (Sigma-Aldrich, St. Louis, MO, USA) and pooled normal human serum from a certified vendor (Innovative Research Inc, Novi, MI, USA) were used in the cultures.

RNA was extracted from each strain using the Direct-zol RNA Kit (Zymo Research, Irvine, CA, USA) following manufacturer’s instructions. Total RNA extractions were performed in three biological replicates for each condition. The extracted and DNase-treated RNA was used to synthesize cDNA using the manufacturer’s protocol provided within the iScriptTM Reverse Transcription Supermix for qPCR (Bio-Rad, Hercules, CA, USA). The cDNA concentrations were adjusted to 50 ng/μL and qPCR was conducted using the qPCRBIO SyGreen Blue Mix Lo-ROX following manufacturer’s protocol (PCR Biosystems, Wayne, PA, USA). At least three biological replicates of cDNA were used in triplets and were run using the CFX96 TouchTM Real-Time PCR Detection System (Bio-Rad, Hercules, CA, USA).

Transcriptional levels of each sample were normalized to the transcriptional level of *rpoB*. The relative quantification of gene expression was performed using the comparative threshold method 2^−ΔΔCt^. The ratios obtained after normalization were expressed as folds of change compared with cDNA samples isolated from bacteria cultures on LB. Asterisks indicate significant differences as determined by ANOVA followed by Tukey’s multiple comparison test (*p* < 0.05), using GraphPad Prism (GraphPad software, San Diego, CA, USA).

### 3.3. Antimicrobial Susceptibility Testing

Antibiotic susceptibility assays were performed following the procedures recommended by the Clinical and Laboratory Standards Institute (CLSI) [51]. After OD adjustment, 100 μL of cells grown in fresh LB medium, 3.5% HSA containing LB medium or HS, were inoculated on Mueller-Hinton agar plates as previously described [17,21]. Antimicrobial commercial E-strips (Liofilchem S.r.l., Roseto degli Abruzzi, Italy) for rifampicin (RIF), tobramycin (TOB), amikacin (AK), and gentamicin (CN) were used. Mueller-Hinton agar plates were incubated at 37 °C for 18 h. CLSI breakpoints were used for interpretation.

### 3.4. Biofilm Assays

Biofilms assays were performed as previously described [21]. *A. baumannii* A118 and AB5075 cells were cultured in fresh LB medium, 3.5% HSA containing LB medium or HS, with agitation for 18 h at 37 °C. Tubes were emptied, washed three times with 1× phosphate-buffered saline (PBS) and stained with 1% crystal violet (CV) for 15 m. Excess CV was removed by washing three more with 1× PBS. Experiments were performed in triplicate, with at least three technical replicates per biological replicate.

### 3.5. N-Acyl Homoserine Lactone (AHL) Detection

*Agrobacterium tumefaciens*-based solid plate assays were carried out to detect AHL production [54] as previously described [55]. Briefly, 500 µL of the homogenate were loaded in a central well of 0.7% LB agar plates supplemented with 40 µg of 5-bromo-3-indolyl-β-D-galactopyranoside (X-Gal) per mL and 250 µL (OD = 2.5) of overnight cultures of *Agrobacterium tumefaciens* biosensor. The presence of AHL was determined by the development of the blue color [56]. As a positive control, 100 µL of N-Decanoyl-DL-homoserine lactone (C10-AHL) 12.5 mg/mL, and as negative control, 100 µL of pooled normal HS was utilized. Quantification of 5,5′-dibromo-4,4′-dichloro-indigo production in different conditions was determined by measuring the intensity of each complete plate and substracting the intensity measured in the negative control, using ImageJ software (NIH). The values were normalized to the positive control, which received the arbitrary value of 100.

### 3.6. Statistical Analysis

All experiments were performed at least in three technical and biological replicates. Data were expressed as means ± standard deviation. Statistical analysis using one way-ANOVA followed by Tukey’s multiple comparison test were performed using GraphPad Prism (GraphPad software, San Diego, CA, USA), and a *p* value < 0.05 was considered statistically significant.

## 4. Conclusions

Our results reveal that in *A. baumannii* a variety of proteins present in human fluids that are encountered during infection significantly modulate a transcriptional response resulting in opportunities for increasing survival. Changes at the transcriptional level affecting genes involved in antimicrobial resistance were observed in the five different *A. baumannii* strains used, despite a differential behavior depending on the studied gene and strain. Results of biofilm formation were in accordance with previous observations obtained with other human fluids [16], where a reduced expression of genes involved in biofilm formation, supported by a substantial decrease in biofilm formation, was observed in all the strains. The transcriptional level of quorum sensing network genes, known to control different virulence factors, was also affected. A reduction in quorum sensing molecules was observed, suggesting a switch towards a commensal state facilitating its persistence. The inhibition of the quorum sensing network is in agreement with the reduced biofilm formation, allowing *A. baumannii* to survive in the bloodstream, where HSA, the main component of HS, is the predominant protein.

Together, the results presented provide important clues that a clear adaptative response of *A. baumannii* is induced by HSA, opening a new venue to explore alternative therapeutic approaches [15,16,17,21,57].

## Figures and Tables

**Table 1:** MICs of A118 or AB5075 grew in LB, LB plus 3.5 % HSA, or HS.

Strain	A118			AB5075		
Antibiotic (μg/mL)	LB	3.5% HSA	HS	LB	3.5% HSA	HS
AK	2 (S)	2 (S)	2 (S)	32 (I)	32 (I)	32 (I)
CN	1 (S)	1 (S)	0.75 (S)	64 (R)	512 (R)	96 (R)
TOB	0.75 (S)	0.75 (S)	0.75 (S)	32 (R)	512 (R)	32 (R)
RIF	2 (S)	1.5 (S)	2 (S)	64 (R)	0.38 (S)	3 (S)
CLO	>256 (R)	>256 (R)	>256 (R)	>256 (R)	>256 (R)	>256 (R)

AK: amikacin; CN: gentamicin; TOB: tobramycin, RIF: rifampicin, CLO: chloramphenicol. R: resistant; I: intermediate; S: susceptible.

## Data Availability

Data is contained within the article.

## Supplementary Materials

The following are available online at https://www.mdpi.com/article/10.3390/antibiotics10070833/s1, Figure S1: Effect of human serum albumin (HSA) and pooled normal human serum (HS) on the expression of antibiotic resistance genes of A. baumannii AMA16, AB0057 and ABUH702 strains, Figure S2: Effect of human serum albumin (HSA) and pooled normal human serum (HS) on the antimicrobial susceptibility of A. baumannii AB5075 strain, Figure S3. Effect of human serum albumin (HSA) and pooled normal human serum (HS) on the antimicrobial susceptibility of A. baumannii AB0057 and ABUH702 strains, and Figure S4: Effect of human serum albumin (HSA) and pooled normal human serum (HS) on biofilm formation in A. baumannii AMA16, AB0057 and ABUH702 strains.

## References

[1] Boucher H.W., Talbot G.H., Bradley J.S., Edwards J.E., Gilbert D., Rice L.B., Scheld M., Spellberg B., Bartlett J. (2009). Bad bugs, no drugs: No ESKAPE! An update from the Infectious Diseases Society of America. Clin. Infect. Dis..

[2] Rice L.B. (2008). Federal funding for the study of antimicrobial resistance in nosocomial pathogens: No ESKAPE. J. Infect. Dis..

[3] Rodriguez C.H., Nastro M., Famiglietti A. (2018). Carbapenemases in *Acinetobacter baumannii*. Review of their dissemination in Latin America. Rev. Argent. Microbiol..

[4] CDC (2019). Antibiotic Resistance Threats in the United States.

[5] Mulani M.S., Kamble E.E., Kumkar S.N., Tawre M.S., Pardesi K.R. (2019). Emerging Strategies to Combat ESKAPE Pathogens in the Era of Antimicrobial Resistance: A Review. Front. Microbiol..

[6] Karaiskos I., Lagou S., Pontikis K., Rapti V., Poulakou G. (2019). The “Old” and the “New” Antibiotics for MDR Gram-Negative Pathogens: For Whom, When, and How. Front. Public Health.

[7] Bassetti M., Echols R., Matsunaga Y., Ariyasu M., Doi Y., Ferrer R., Lodise T.P., Naas T., Niki Y., Paterson D.L. (2021). Efficacy and safety of cefiderocol or best available therapy for the treatment of serious infections caused by carbapenem-resistant Gram-negative bacteria (CREDIBLE-CR): A randomised, open-label, multicentre, pathogen-focused, descriptive, phase 3 trial. Lancet Infect. Dis..

[8] Bassetti M., Peghin M., Vena A., Giacobbe D.R. (2019). Treatment of Infections Due to MDR Gram-Negative Bacteria. Front. Med..

[9] Bulens S.N., Yi S.H., Walters M.S., Jacob J.T., Bower C., Reno J., Wilson L., Vaeth E., Bamberg W., Janelle S.J. (2018). Carbapenem-Nonsusceptible *Acinetobacter baumannii*, 8 US Metropolitan Areas, 2012–2015. Emerg. Infect. Dis..

[10] Isler B., Doi Y., Bonomo R.A., Paterson D.L. (2019). New treatment options against carbapenem-resistant *Acinetobacter baumannii* infections. Antimicrob. Agents Chemother..

[11] Paterson D.L., Isler B., Stewart A. (2020). New treatment options for multiresistant gram negatives. Curr. Opin. Infect. Dis..

[12] Wong D., Nielsen T.B., Bonomo R.A., Pantapalangkoor P., Luna B., Spellberg B. (2017). Clinical and Pathophysiological Overview of *Acinetobacter* Infections: A Century of Challenges. Clin. Microbiol. Rev..

[13] Muller G.L., Tuttobene M., Altilio M., Martinez Amezaga M., Nguyen M., Cribb P., Cybulski L.E., Ramirez M.S., Altabe S., Mussi M.A. (2017). Light Modulates Metabolic Pathways and Other Novel Physiological Traits in the Human Pathogen *Acinetobacter baumannii*. J. Bacteriol..

[14] Tuttobene M.R., Fernandez-Garcia L., Blasco L., Cribb P., Ambroa A., Muller G.L., Fernandez-Cuenca F., Bleriot I., Rodriguez R.E., Barbosa B.G.V. (2019). Quorum and Light Signals Modulate Acetoin/Butanediol Catabolism in *Acinetobacter* spp.. Front. Microbiol..

[15] Martinez J., Razo-Gutierrez C., Le C., Courville R., Pimentel C., Liu C., Fung S.E., Tuttobene M.R., Phan K., Vila A.J. (2021). Cerebrospinal fluid (CSF) augments metabolism and virulence expression factors in *Acinetobacter baumannii*. Sci. Rep..

[16] Pimentel C., Le C., Tuttobene M.R., Subils T., Martinez J., Sieira R., Papp-Wallace K.M., Keppetipola N., Bonomo R.A., Actis L.A. (2021). Human Pleural Fluid and Human Serum Albumin Modulate the Behavior of a Hypervirulent and Multidrug-Resistant (MDR) *Acinetobacter baumannii* Representative Strain. Pathogens.

[17] Martinez J., Fernandez J.S., Liu C., Hoard A., Mendoza A., Nakanouchi J., Rodman N., Courville R., Tuttobene M.R., Lopez C. (2019). Human pleural fluid triggers global changes in the transcriptional landscape of *Acinetobacter baumannii* as an adaptive response to stress. Sci. Rep..

[18] Alvarez-Fraga L., Vazquez-Ucha J.C., Martinez-Guitian M., Vallejo J.A., Bou G., Beceiro A., Poza M. (2018). Pneumonia infection in mice reveals the involvement of the feoA gene in the pathogenesis of *Acinetobacter baumannii*. Virulence.

[19] Lopez M., Blasco L., Gato E., Perez A., Fernandez-Garcia L., Martinez-Martinez L., Fernandez-Cuenca F., Rodriguez-Bano J., Pascual A., Bou G. (2017). Response to Bile Salts in Clinical Strains of *Acinetobacter baumannii* Lacking the AdeABC Efflux Pump: Virulence Associated with Quorum Sensing. Front. Cell Infect. Microbiol..

[20] Rodman N., Martinez J., Fung S., Nakanouchi J., Myers A.L., Harris C.M., Dang E., Fernandez J.S., Liu C., Mendoza A.M. (2019). Human Pleural Fluid Elicits Pyruvate and Phenylalanine Metabolism in *Acinetobacter baumannii* to Enhance Cytotoxicity and Immune Evasion. Front. Microbiol..

[21] Quinn B., Rodman N., Jara E., Fernandez J.S., Martinez J., Traglia G.M., Montana S., Cantera V., Place K., Bonomo R.A. (2018). Human serum albumin alters specific genes that can play a role in survival and persistence in *Acinetobacter baumannii*. Sci. Rep..

[22] Ohneck E.J., Arivett B.A., Fiester S.E., Wood C.R., Metz M.L., Simeone G.M., Actis L.A. (2018). Mucin acts as a nutrient source and a signal for the differential expression of genes coding for cellular processes and virulence factors in *Acinetobacter baumannii*. PLoS ONE.

[23] Qin H., Lo N.W., Loo J.F., Lin X., Yim A.K., Tsui S.K., Lau T.C., Ip M., Chan T.F. (2018). Comparative transcriptomics of multidrug-resistant *Acinetobacter baumannii* in response to antibiotic treatments. Sci. Rep..

[24] Quinn B., Martinez J., Liu C., Nguyen M., Ramirez M.S. (2018). The effect of sub-inhibitory concentrations of antibiotics on natural transformation in *Acinetobacter baumannii*. Int. J. Antimicrob. Agents.

[25] Sarshar M., Behzadi P., Scribano D., Palamara A.T., Ambrosi C. (2021). *Acinetobacter baumannii*: An Ancient Commensal with Weapons of a Pathogen. Pathogens.

[26] Karalewitz A.P., Miller S.I. (2018). Multidrug-Resistant *Acinetobacter baumannii* Chloramphenicol Resistance Requires an Inner Membrane Permease. Antimicrob. Agents Chemother..

[27] Vashist J., Tiwari V., Das R., Kapil A., Rajeswari M.R. (2011). Analysis of penicillin-binding proteins (PBPs) in carbapenem resistant *Acinetobacter baumannii*. Indian J. Med. Res..

[28] Fernandez-Cuenca F., Martinez-Martinez L., Conejo M.C., Ayala J.A., Perea E.J., Pascual A. (2003). Relationship between beta-lactamase production, outer membrane protein and penicillin-binding protein profiles on the activity of carbapenems against clinical isolates of *Acinetobacter baumannii*. J. Antimicrob. Chemother..

[29] Ramirez M.S., Tolmasky M.E. (2010). Aminoglycoside modifying enzymes. Drug Resist. Updat..

[30] Hong S.B., Shin K.S., Ha J., Han K. (2013). Co-existence of blaOXA-23 and armA in multidrug-resistant *Acinetobacter baumannii* isolated from a hospital in South Korea. J. Med. Microbiol..

[31] Park H.J., Cho J.H., Kim H.J., Han S.H., Jeong S.H., Byun M.K. (2019). Colistin monotherapy versus colistin/rifampicin combination therapy in pneumonia caused by colistin-resistant *Acinetobacter baumannii*: A randomised controlled trial. J. Glob. Antimicrob. Resist..

[32] Dominguez M., Miranda C.D., Fuentes O., de la Fuente M., Godoy F.A., Bello-Toledo H., Gonzalez-Rocha G. (2019). Occurrence of Transferable Integrons and sul and dfr Genes Among Sulfonamide-and/or Trimethoprim-Resistant Bacteria Isolated From Chilean Salmonid Farms. Front. Microbiol..

[33] Ozkul C., Hazirolan G. (2020). Oxacillinase Gene Distribution, Antibiotic Resistance, and Their Correlation with Biofilm Formation in *Acinetobacter baumannii* Bloodstream Isolates. Microb. Drug Resist..

[34] Huang J.X., Blaskovich M.A., Pelingon R., Ramu S., Kavanagh A., Elliott A.G., Butler M.S., Montgomery A.B., Cooper M.A. (2015). Mucin Binding Reduces Colistin Antimicrobial Activity. Antimicrob. Agents Chemother..

[35] Hassan P.A., Khider A.K. (2020). Correlation of biofilm formation and antibiotic resistance among clinical and soil isolates of *Acinetobacter baumannii* in Iraq. Acta Microbiol. Immunol. Hung..

[36] Qi L., Li H., Zhang C., Liang B., Li J., Wang L., Du X., Liu X., Qiu S., Song H. (2016). Relationship between Antibiotic Resistance, Biofilm Formation, and Biofilm-Specific Resistance in *Acinetobacter baumannii*. Front. Microbiol..

[37] Senobar Tahaei S.A., Stajer A., Barrak I., Ostorhazi E., Szabo D., Gajdacs M. (2021). Correlation Between Biofilm-Formation and the Antibiotic Resistant Phenotype in *Staphylococcus aureus* Isolates: A Laboratory-Based Study in Hungary and a Review of the Literature. Infect. Drug Resist..

[38] Tomaras A.P., Flagler M.J., Dorsey C.W., Gaddy J.A., Actis L.A. (2008). Characterization of a two-component regulatory system from *Acinetobacter baumannii* that controls biofilm formation and cellular morphology. Microbiology.

[39] Gaddy J.A., Tomaras A.P., Actis L.A. (2009). The *Acinetobacter baumannii* 19606 OmpA protein plays a role in biofilm formation on abiotic surfaces and in the interaction of this pathogen with eukaryotic cells. Infect. Immun..

[40] Vijayakumar S., Rajenderan S., Laishram S., Anandan S., Balaji V., Biswas I. (2016). Biofilm Formation and Motility Depend on the Nature of the *Acinetobacter baumannii* Clinical Isolates. Front. Public Health.

[41] Tiwari V., Patel V., Tiwari M. (2018). In-silico screening and experimental validation reveal L-Adrenaline as anti-biofilm molecule against biofilm-associated protein (Bap) producing *Acinetobacter baumannii*. Int. J. Biol. Macromol..

[42] Avery T.M., Boone R.L., Lu J., Spicer S.K., Guevara M.A., Moore R.E., Chambers S.A., Manning S.D., Dent L., Marshall D. (2021). Analysis of Antimicrobial and Antibiofilm Activity of Human Milk Lactoferrin Compared to Bovine Lactoferrin against Multidrug Resistant and Susceptible *Acinetobacter baumannii* Clinical Isolates. ACS Infect. Dis..

[43] Camilli A., Bassler B.L. (2006). Bacterial small-molecule signaling pathways. Science.

[44] Seleem N.M., Abd El Latif H.K., Shaldam M.A., El-Ganiny A. (2020). Drugs with new lease of life as quorum sensing inhibitors: For combating MDR *Acinetobacter baumannii* infections. Eur. J. Clin. Microbiol. Infect. Dis..

[45] Saipriya K., Swathi C.H., Ratnakar K.S., Sritharan V. (2020). Quorum-sensing system in *Acinetobacter baumannii*: A potential target for new drug development. J. Appl. Microbiol..

[46] Lopez M., Mayer C., Fernandez-Garcia L., Blasco L., Muras A., Ruiz F.M., Bou G., Otero A., Tomas M., Geih G. (2017). Quorum sensing network in clinical strains of *A. baumannii*: AidA is a new quorum quenching enzyme. PLoS ONE.

[47] Lopez-Martin M., Dubern J.F., Alexander M.R., Williams P. (2021). AbaM Regulates Quorum Sensing, Biofilm Formation and Virulence in *Acinetobacter baumannii*. J. Bacteriol..

[48] Eze E.C., Chenia H.Y., El Zowalaty M.E. (2018). *Acinetobacter baumannii* biofilms: Effects of physicochemical factors, virulence, antibiotic resistance determinants, gene regulation, and future antimicrobial treatments. Infect. Drug Resist..

[49] Niu C., Clemmer K.M., Bonomo R.A., Rather P.N. (2008). Isolation and characterization of an autoinducer synthase from *Acinetobacter baumannii*. J. Bacteriol..

[50] Adams M.D., Goglin K., Molyneaux N., Hujer K.M., Lavender H., Jamison J.J., MacDonald I.J., Martin K.M., Russo T., Campagnari A.A. (2008). Comparative genome sequence analysis of multidrug-resistant *Acinetobacter baumannii*. J. Bacteriol..

[51] C.L.S.I. (2019). Performance Standards for Antimicrobial Susceptibility Testing: Twenty-Nine Informational Supplement.

[52] Adams M.D., Pasteran F., Traglia G.M., Martinez J., Huang F., Liu C., Fernandez J.S., Lopez C., Gonzalez L.J., Albornoz E. (2020). Distinct mechanisms of dissemination of NDM-1 metallo- beta-lactamase in *Acinetobacter* spp. in Argentina. Antimicrob. Agents Chemother..

[53] Adams M.D., Wright M.S., Karichu J.K., Venepally P., Fouts D.E., Chan A.P., Richter S.S., Jacobs M.R., Bonomo R.A. (2019). Rapid Replacement of *Acinetobacter baumannii* Strains Accompanied by Changes in Lipooligosaccharide Loci and Resistance Gene Repertoire. mBio.

[54] Paulk Tierney A.R., Rather P.N. (2019). Methods for Detecting N-Acyl Homoserine Lactone Production in *Acinetobacter baumannii*. Methods Mol. Biol..

[55] Pimentel C.B., Snow A.L., Carnes S.L., Shah N.R., Loup J.R., Vallejo-Luces T.M., Madrigal C., Hartmann C.W. (2021). Huddles and their effectiveness at the frontlines of clinical care: A scoping review. J. Gen. Intern. Med..

[56] Cha C., Gao P., Chen Y.C., Shaw P.D., Farrand S.K. (1998). Production of acyl-homoserine lactone quorum-sensing signals by gram-negative plant-associated bacteria. Mol. Plant. Microbe. Interact..

[57] Martinez J., Liu C., Rodman N., Fernandez J.S., Barberis C., Sieira R., Perez F., Bonomo R.A., Ramirez M.S. (2018). Human fluids alter DNA-acquisition in *Acinetobacter baumannii*. Diagn. Microbiol. Infect. Dis..

