# Supplementary material for: Interaction of Acinetobacter baumannii with Human Serum Albumin: Does the Host Determine the Outcome?
Source: Antibiotics (Basel). 2021 Jul 8;10(7):833. doi: 10.3390/antibiotics10070833 (PMC8300715; doi:10.3390/antibiotics10070833)
Supplement: Supplementary file 1 [file antibiotics-10-00833-s001.zip › antibiotics-1266400-supplementary.pdf]

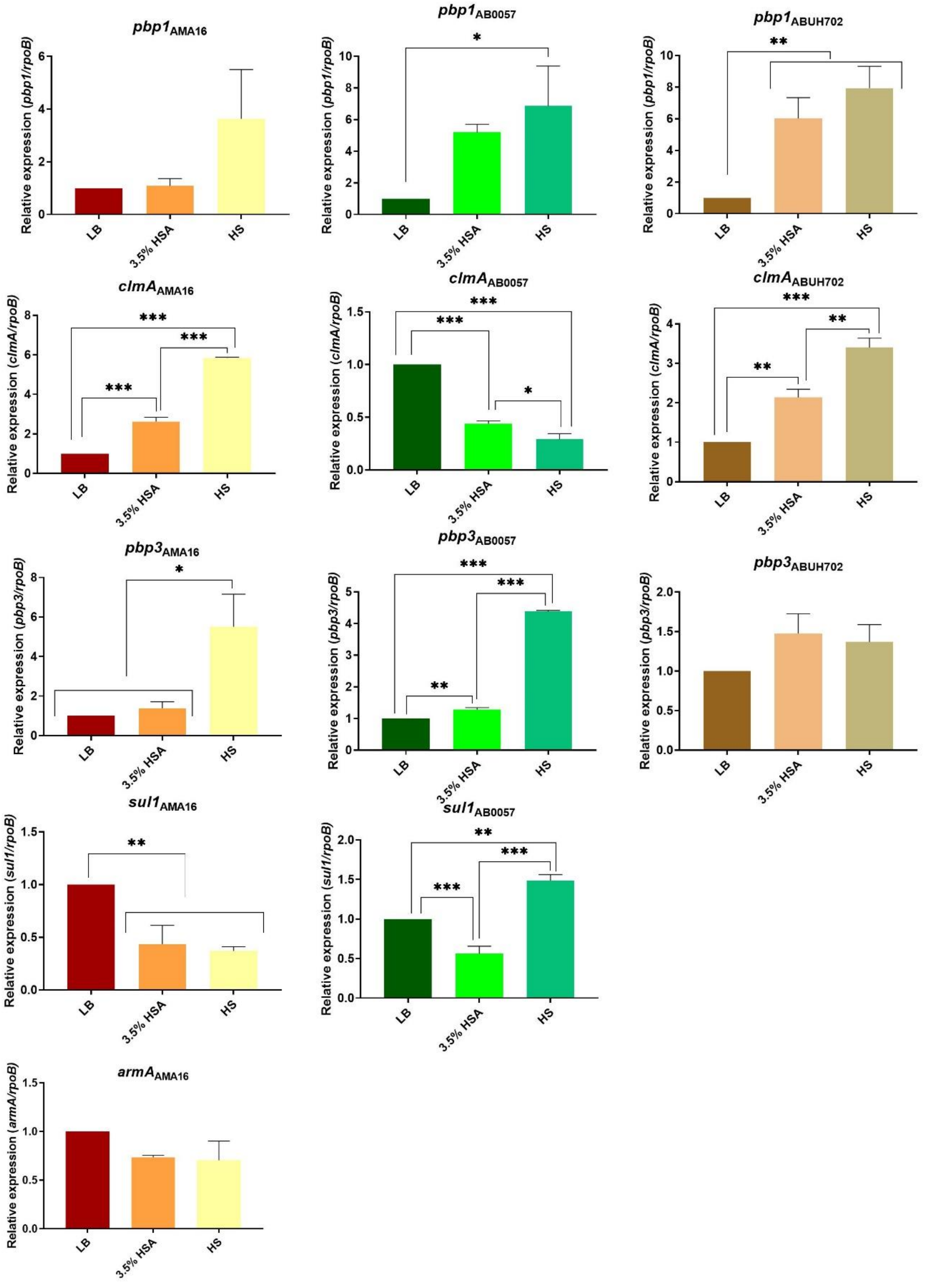

Figure S1. Effect of human serum albumin (HSA) and pooled normal human serum (HS) on the expression of antibiotic resistance genes of *A. baumannii* AMA16, AB0057 and ABUH702 strains**.** qRT-PCR of genes associated with antibiotic resistance expressed in LB, LB supplemented with 3.5 % HSA or in HS. Fold changes were calculated using double ΔΔCt analysis. At least three independent samples were used. LB was used as the reference condition. Statistical significance (p < 0.05) was determined by ANOVA followed by Tukey's multiple-comparison test, one asterisks: $p < 0.05$; two asterisks: p < 0.01 and three asterisks: $p < 0.001$.

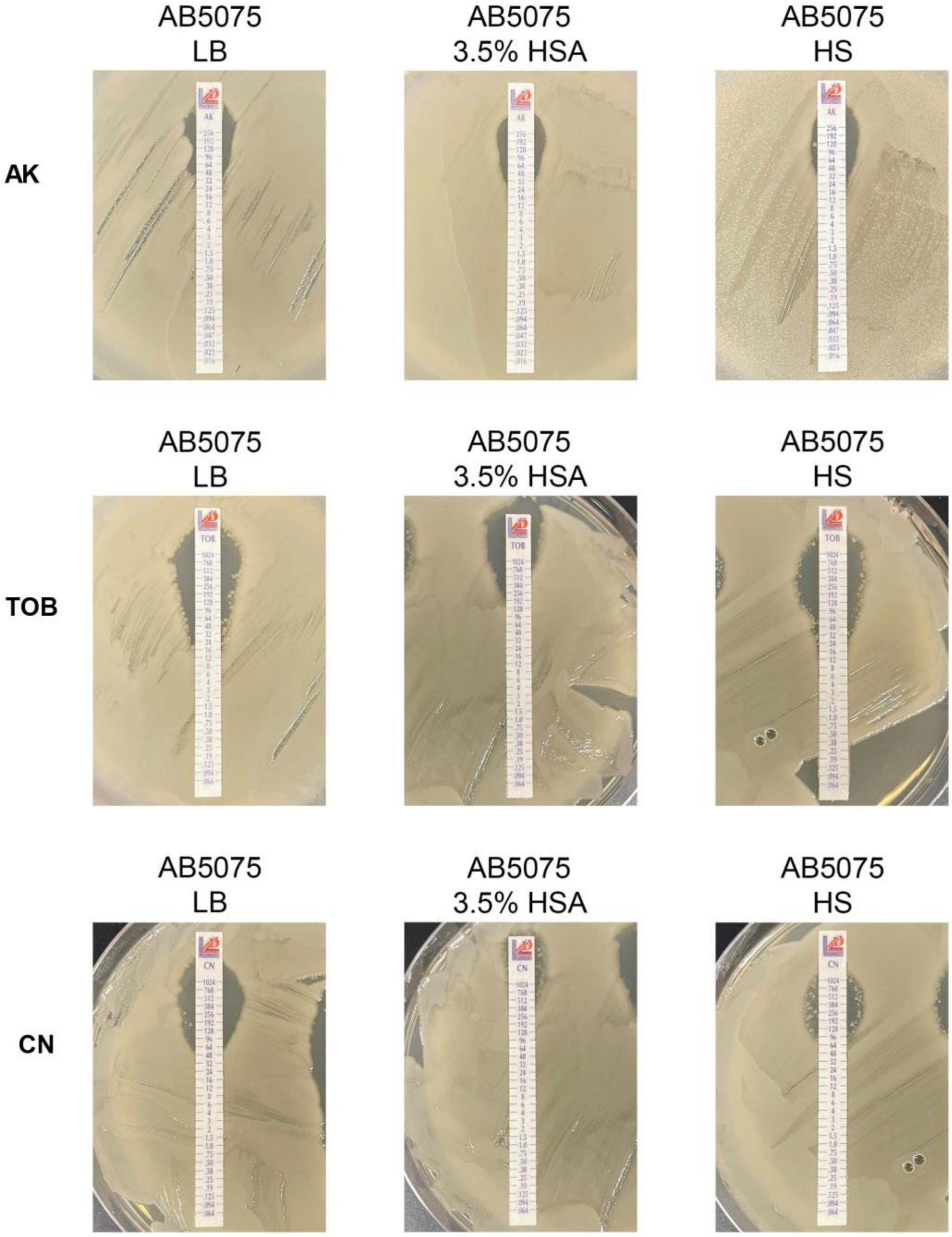

Figure S2. Effect of human serum albumin (HSA) and pooled normal human serum (HS) on the antimicrobial susceptibility of *A. baumannii* AB5075 strain grew in LB broth, LB broth plus 3.5 % HSA or in HS were used to performed amikacin (AK), tobramycin (TOB) and gentamicin (CN) susceptibility. Minimum inhibitory concentration (MIC) was performed by E-test (Liofilchem, Italy) following CLSI recommendations.

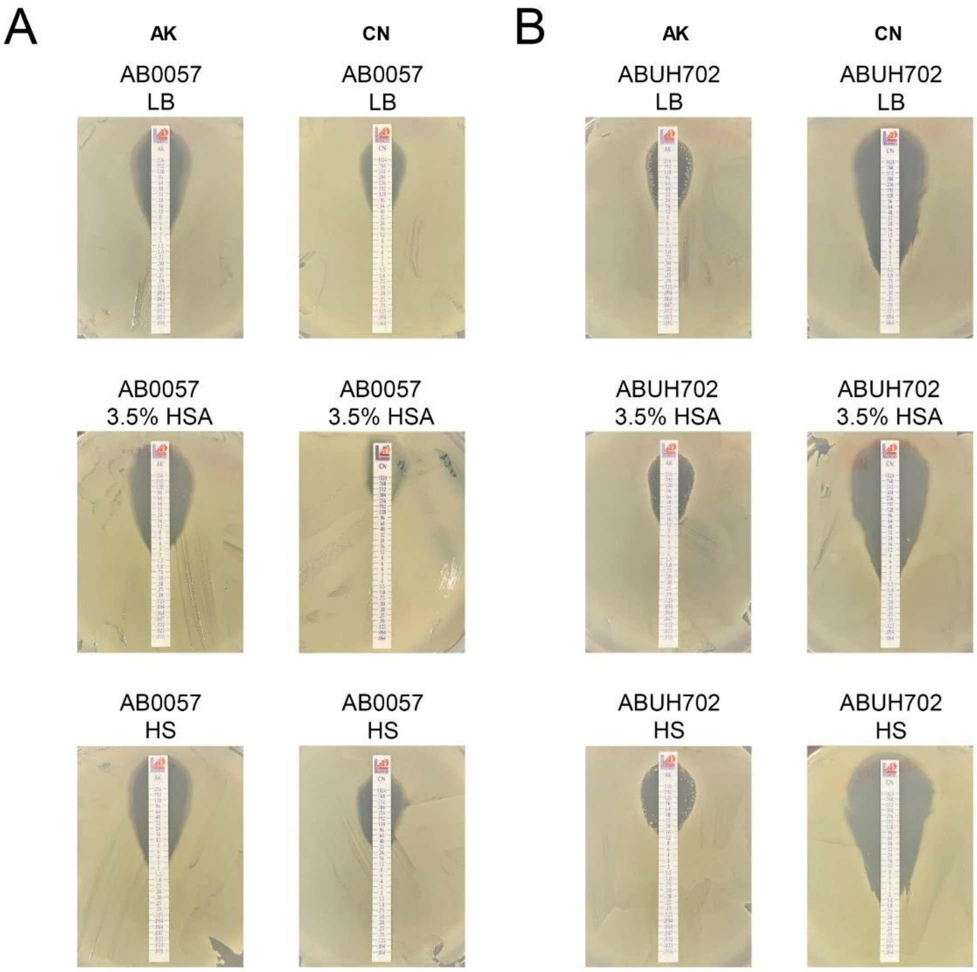

Figure S3. Effect of human serum albumin (HSA) and pooled normal human serum (HS) on the antimicrobial susceptibility of *A. baumannii* AB0057 and ABUH702 strains grew in LB broth, LB broth plus 3.5 % HSA or in HS were used to performed amikacin (AK) and gentamicin (CN) susceptibility. Minimum inhibitory concentration (MIC) was performed by E-test (Liofilchem, Italy) following CLSI recommendations.

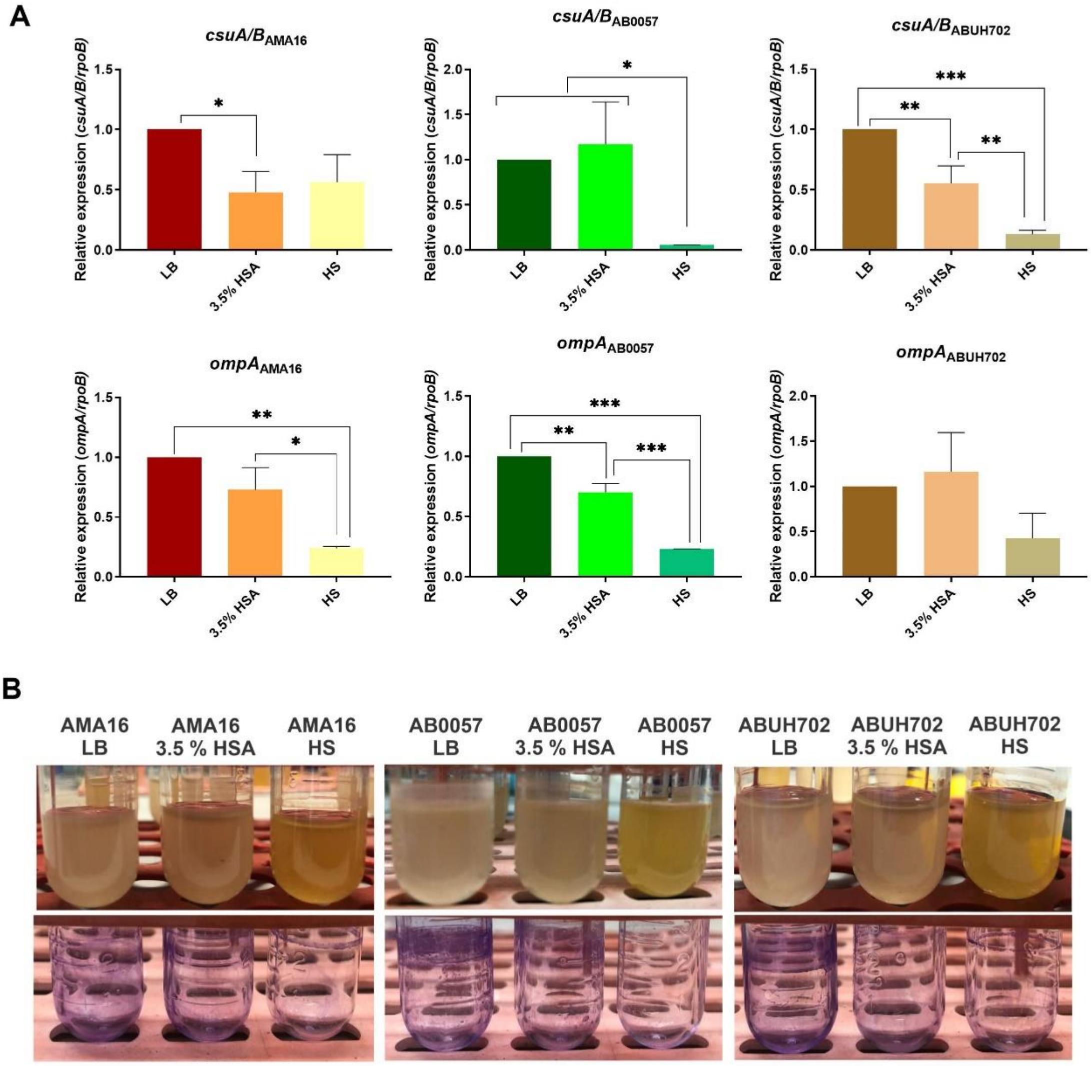

Figure S4. Effect of human serum albumin (HSA) and pooled normal human serum (HS) on biofilm formation in *A. buamannii* AMA16, AB0057 and ABUH702 strains. (A) qRT-PCR of AMA16, AB0057 and ABUH702 strains of the *csuA/B* and *ompA* genes biofilm formation related in cells cultured in LB, LB supplemented with 3.5 % HSA or in HS. Fold changes were calculated using double ΔΔCt analysis. At least three independent samples were used. LB was used as the reference condition. Statistical significance ($p < 0.05$) was determined by ANOVA followed by Tukey's multiple-comparison test, one asterisks: $p < 0.05$; two asterisks: $p < 0.01$ and three asterisks: $p < 0.001$. (B) Biofilm assays performed in AMA16, AB0057 and ABUH702 strains grown in LB, LB supplemented with 3.5 % HSA or in HS. Tubes were stained with 1% crystal violet.
